# Caspase-14: A novel caspase in the retina with a potential role in diabetic retinopathy

**Published:** 2012-07-14

**Authors:** Mohamed Al-Shabrawey, Saif Ahmad, Sylvia Megyerdi, Amira Othman, Babak Baban, Tammy L. Palenski, Eui Seok Shin, Zafer Gurel, Stephen Hsu, Nader Sheibani

**Affiliations:** 1 Department of Oral Biology and Anatomy, Georgia Health Sciences University (GHSU), College of Dental Medicine, Augusta, GA; 2 Ophthalmology and Vision Discovery Institute, GHSU, Medical College of Georgia, Augusta, GA; 3 Department of Anatomy, Mansoura Faculty of Medicine, Mansoura, Egypt; 4 Ophthalmology and Visual Sciences, University of Wisconsin School of Medicine and Public Health, Madison, WI

## Abstract

**Purpose:**

The purpose of this study was to evaluate caspase-14 expression in the retina under normal and diabetic conditions, and to determine whether caspase-14 contributes to retinal microvascular cell death under high glucose conditions.

**Methods:**

Quantitative real-time polymerase chain reaction and western blot analysis were used to evaluate caspase-14 expression in retinal cells, including pericytes (PCs), endothelial cells (ECs), astrocytes (ACs), choroidal ECs, and retinal pigment epithelium (RPE) cells. We also determined caspase-14 expression in the retinas of human subjects with or without diabetic retinopathy (DR) and in experimental diabetic mice. Retinal ECs and PCs were infected with adenoviruses expressing human caspase-14 or green fluorescent protein. Caspase-14 expression was also assessed in retinal vascular cells cultured under high glucose conditions. The number of apoptotic cells was determined with terminal deoxynucleotidyl transferase dUTP nick end labeling staining and confirmed by determining the levels of cleaved poly (ADP-ribose) polymerase-1 and caspase-3.

**Results:**

Our experiments demonstrated that retinal ECs, PCs, ACs, choroidal ECs, and RPE cells expressed caspase-14, and DR was associated with upregulation and/or activation of caspase-14 particularly in retinal vasculature. High glucose induced marked elevation of the caspase-14 level in retinal vascular cells. There was a significant increase in the apoptosis rate and the levels of cleaved poly (ADP-ribose) polymerase-1 and caspase-3 in retinal ECs and PCs overexpressing caspase-14.

**Conclusions:**

Our findings indicate that caspase-14 might play a significant role in the pathogenesis of DR by accelerating retinal PC and EC death. Further investigations are required to elaborate the underlying mechanisms.

## Introduction

Diabetic retinopathy (DR) is a major cause of blindness in the United States [1,2]. Early inflammatory responses, disruption of the blood–retinal barrier, accelerated microvascular cell death, and pathological angiogenesis are all hallmarks of DR. Hyperglycemia triggers capillary degeneration leading to disruption of the blood–retinal barrier and the formation of acellular capillaries and subsequent retinal ischemia and retinal neovascularization [3-7]. Thus, accelerated microvascular cell death is a potential therapeutic target for preventing the development and progression of DR.

Caspases may play crucial roles in the development of DR via inducing and executing the apoptotic cell death program. Caspases are cysteine aspartate proteases that cleave proteins after an aspartate residue [8,9]. According to biologic function, caspases have been classified into different groups, a group specialized in inducing and executing apoptotic cell death (caspase-2, -3, -6, -7, -8, -9, and -10) and a group that contributes to the intracellular activation of the proinflammatory cytokines interleukin (IL)-1β and IL-18 (caspase-1, -4, -5, and -11) [8,10]. A third group of caspases is involved mainly in epithelial differentiation, in particular terminal differentiation of keratinocytes (caspase-14) [11]. Caspase-14 is expressed and activated mainly in the epidermis and is not detected in most other adult tissues [11]. Caspase-14 is found in tissues involved in barrier function such as the epidermis, choroid plexus, hair follicles, retinal pigment epithelium, thymic Hassall's bodies, and keratinized oral epithelium [12]. Recently, a recent report showed caspase-14 was expressed in cardiomyocytes and involved in cardiac cell death [13].

The physiologic function of caspase-14 is relatively unexplored. Caspase-14 is thought to be mainly associated with terminal differentiation of normal human epidermal keratinocytes and epidermal barrier formation that protects against dehydration and ultraviolet B radiation-induced apoptosis. Caspase-14 has substrate specificity similar to the cytokine activator caspases. Similar to other procaspases, caspase-14 also requires proteolytic processing within its catalytic domain before the competent enzyme is dimerized and generated [14]. The substrate preferences for human and mouse enzymes are different, with data suggesting that human caspase-14 is comparable to cytokine activator caspases-1, -4, and -5, while mouse caspase-14 is more comparable to initiator caspases-8 and -9 [14]. Nevertheless, caspase-14 expression and function in the retina has not yet been explored. Information regarding caspase-14 expression and function in the ocular tissue, and more specifically in the retina, is lacking. A recent study demonstrated a marked increase in the amount of caspase-14 present in aqueous humor of patients with glaucoma [15], supporting the presence of caspase-14 in the eye and its potential role in ocular diseases including DR.

Development of retinal acellular capillaries, due to accelerated apoptosis of microvascular cells during diabetes, results in ischemia that then promotes neovascularization in an attempt to restore blood flow [16]. The newly formed vessels destroy the normal retinal architecture and are leaky resulting in impaired vision [17]. In addition to vascular endothelial cells (ECs), pericyte (PC) loss is considered a hallmark of early DR and contributes to the development of retinal acellular capillaries and subsequent retinal neovascularization [18-20]. The mechanisms by which diabetes influences apoptosis of the retinal microvasculature are not yet fully understood. However, many factors have been suggested to be involved, such as oxidative stress, formation of advanced glycation end products, upregulation of protein kinase C (PKC), and increased polyol pathway flux [21]. We hypothesized that enhanced expression of caspase-14 promotes the accelerated death of microvascular cells during diabetes.

Here we examined the expression of caspase-14 in the retina and various retinal cells. We also determined the impact of diabetes or high glucose on caspase-14 expression in the retinas of human subjects and mice, as well as cultured retinal microvascular cells. In addition, we tested whether increased caspase-14 expression in retinal ECs and PCs results in an increased rate of apoptosis and expression of the apoptotic markers. These studies demonstrated, for the first time, that caspase-14 is expressed in the retina and different retinal cells under normal conditions, and increased expression of caspase-14 occurs in the retinas of diabetic human subjects and experimental mice, as well as in retinal microvascular cells cultured under high glucose conditions.

## Methods

### Human tissues

Human retina and retinal sections were obtained from the Cooperative Human Tissue Network Hospital (CHTN) of the University of Pennsylvania and Capital Bioscience (Rockville, MD).

### Animals

C57BL/6J mice from Jackson Labs (Bar Harbor, ME) were used according to the Association for Research in Vision and Ophthalmology Statement for the Use of Animals in Ophthalmic and Vision Research and approved by Institutional Animal Care and Use Committee. Experimental diabetes was developed in one group by intraperitoneal injection of streptozotocin (65 mg/kg) dissolved in distilled water. The mean glucose blood level was 437±53. Six weeks after the onset of diabetes, one retina from each animal was immediately frozen in liquid nitrogen and stored at −80 °C for western blot analysis, and the other eyeball was embedded in optical coherence tomography for sectioning and immunohistochemistry.

### Cell culture

The primary mouse retinal cells, including PCs, ECs, astrocytes (ACs), retinal pigment epithelium (RPE) cells, choroidal endothelial cells (ChECs), trabecular meshwork (TM) cells, and lung ECs were prepared from C57BL/6J immortomice and cultured as previously described [22]. For high glucose studies, the cells were cultured in growth medium containing 5.5 mM (normal, NG), 40.5 mM (high, HG), 5.5 mM D-glucose, and 35 mM L-glucose (osmolarity control) for five days before analysis [23]. The cells were fed with fresh medium every other day. Bovine retinal endothelial cells (BRECs) were prepared as previously described [24]. Briefly, retinas were removed from bovine eyes then, homogenized (2–3 strokes by Dounce homogenizer) in PBS containing calcium and magnesium, and the resulting homogenate was filtered over an 80 μm nylon sieve. PBS (Gibco, Grand Island, NY) made of potassium phosphate monobasic (KH_2_PO_4_, 10.59 mM), sodium chloride (NaCl, 1551.72 mM and sodium phosphate bibasic, Na_2_HPO_4_-7H_2_O, 29.66 mM). The material retained by the filter was incubated for 45 min at 37 °C in a solution of PBS containing 100 U/ml collagenase and 0.033% BSA (BSA) then, centrifuged at 1,000 ×g for 3 min. The pellet was then, resuspended in Dulbecco's odification of Eagle's medium (DMEM) containing 20% FBS and seeded in dishes precoated with fibronectin/ hyaluronic acid mix (100 mg/ml each in PBS). Cells were allowed to attach for 3 h followed by rinsing with PBS, then covering the cells with serum-defined medium (EGM; Clonetics, San Diego, CA), followed by incubation for a week. To eliminate non-endothelial contaminating cells cultures were covered with serum-free basal medium (EBM or M-199) supplemented with platelet-free serum for 6–7 days and then subcultured into gelatin-coated dishes in M-199 1 10% FBS or serum-defined EGM medium.

### Western blot analysis

We evaluated the expression of caspase-14, poly (ADP-ribose) polymerase-1 (PARP-1), and cleaved caspase-3 in the retinal cells and in the retinas of normal and diabetic mice, as well as human subjects. Briefly, retinal cells or retinas from each mouse and human subject in different groups were homogenized in a modified radioimmunoprecipitation assay buffer (20 mM Tris-HCl [pH 7.4], 2.5 mM EDTA, 50 mM NaF, 10 mM sodium pyrophosphate (Na_2_P_2_O_7_), 1% Triton X-100, 0.1% sodium dodecyl sulfate, 1% sodium deoxycholate, and 1 mM phenylmethylsulfonyl fluoride). Homogenates (50 µg protein) were separated with sodium dodecyl sulfate PAGE using 10% ready precast gel (Bio-Rad, Hercules, CA), transferred to polyvinylidene fluoride membrane, and reacted with rabbit polyclonal caspase-14 antibody (Sc-5628; Santa Cruz Biotechnology, Santa Cruz, CA), or antibodies against PARP-1 (Santa Cruz Biotechnology) and cleaved caspase-3 (Cell Signaling, Danvers, MA) followed by horseradish peroxidase-linked secondary antibody and enhanced chemiluminescence (Amersham Pharmacia, San Francisco, CA). To demonstrate cleaved caspase-14 in different retinal cells, caspase-14 was processed under reduced conditions in the presence of β-mercaptoethanol. The membranes were then stripped and reprobed with β-actin to demonstrate equal loading, and the results were analyzed using the ImageJ program.

### Caspase-14 immunohistochemical analysis

Retinal paraffin sections of human subjects with or without DR were fixed in 10% neutral buffered formalin (HT50–1–128; Sigma, St Louis, MO). Following rehydration of the paraffin section and two washes in PBS, endogenous peroxidase activity was blocked using hydrogen peroxide diluted 1:10 with distilled water for 10 min. Sections were treated with Proteinase K (S3020; Dako, Carpinteria, CA) for 10 min and washed twice in PBS. All preparations were then treated with universal blocking reagent (HK085–5K; Biogenex, Fremont CA) for 8 min according to the manufacturer’s instructions. Excess reagent was removed with a quick rinse with PBS. Sections were incubated with rabbit polyclonal caspase-14 antibody (1:100 dilution in PBS) for 2 h. Following two washes in PBS, biotinylated antirabbit immunoglobulins (HK336–9R; Biogenex) were added to all slides for 30 min, and then peroxidase-conjugated streptavidin (HK330–9K; Biogenex) was added for an additional 30 min. Excess reagent was removed, and the slides were washed in PBS and incubated with chromogen (liquid AEC; HK121–5K; Biogenex) for 10-30 min until the desired color appeared. All preparations were counterstained with hematoxylin (7221; Richard-Allan Scientific, Kalamazoo, MI) for 30 s and mounted in aqueous mounting medium (Faramount aqueous S3025; Dako).

### Caspase-14 immunofluorescence

The expression of caspase-14 in the mouse retina was examined with immunofluorescence using caspase-14 antibody (Santa Cruz Biotechnology) and isolectin-B4 as a vascular marker (Vector Laboratories, Burlingame, CA). Briefly, frozen retinal sections from normal and diabetic mice were fixed in paraformaldehyde (4%) for 10 min followed by incubation in 3% normal goat serum for 1 h. The sections were then incubated in primary caspase-14 antibody (1:100) and isolectin-B4 (15 μg/ml) overnight at 4 °C, followed by avidin-conjugated Texas red (Vector Laboratories) and Oregon green-labeled antirabbit antibody (Molecular Probes, Eugene, OR) to identify the localization of caspase-14 in the retinal sections. Sections were covered using 4’,6 diamidino-2-phenylindole (DAPI) mounting medium (Vector Laboratories), and images were obtained with confocal microscopy (LSM 510; Carl Zeiss, Thornwood, NY).

### RNA purification and quantitative real-time polymerase chain reaction

The total RNA from retinal cells was extracted with the mirVana PARIS kit (Ambion, Grand Island, NY) according to the manufacturer’s instructions. cDNA synthesis was performed from 1 μg of total RNA using the Sprint RT Complete-Double PrePrimed kit (Clontech, Mountain View, CA). One μl of each cDNA (dilution 1:10) was used as the template in the quantitative real-time polymerase chain reaction (qPCR) assays, performed in triplicate of three biologic replicates on Mastercycler RealPlex (Eppendorf, Hauppauge, NY) using the SYBR qPCR Premix (Clontech). Amplification parameters were as follows: 95 °C for 2 min; 40 cycles of amplification (95 °C for 15 s, 60 °C for 40 s); dissociation curve step (95 °C for 15 s, 60 °C for 15 s, 95 °C for 15 s). The list of primers used is shown in Table 1.

**Table 1:** Primer sequences

**Gene name**	**Accession number**	**Forward 5′ to 3′**	**Reverse 5′ to 3′**
Caspase14	NM_009809.5	TGACGCTGTGTGTCACCAAA	GTTCCAGGGCCTCCATGTCT
*RpL13A*	NM_009438.4	TCTCAAGGTTGTTCGGCTGAA	GCCAGACGCCCCAGGTA

Standard curves were generated from known quantities for each target gene of linearized plasmid DNA. Ten times dilution series were used for each known target, which were amplified using SYBR-Green qPCR. The linear regression line for ng of DNA was determined from the relative fluorescent units at a threshold fluorescence value to quantify gene targets from cell extracts by comparing the relative fluorescent units at the threshold fluorescence value to the standard curve, normalized by the simultaneous amplification of ribosomal protein L13a (*Rpl13A*), which was used as a housekeeping gene to normalize all samples.

### Adenovirus-mediated expression of caspase-14

We constructed a caspase-14-expression adenovirus encoding the full-length human caspase-14 cDNA and green fluorescent protein (*GFP*) cDNA using the AdEasy system (Agilent Technologies, La Jolla, CA) as previously described [25]. The control adenoviral vector encoded only the *GFP* cDNA. Infection was performed according to the result of the infection titration. Expression of caspase-14 was confirmed with western blotting.

### Caspase-14 transfection

To avoid the overlap between the GFP and green reaction in our terminal deoxynucleotidyl transferase dUTP nick end labeling (TUNEL) assay of retinal endothelial cell apoptosis, we overexpressed caspase-14 using transfection method. BRECs were transfected with the pCMV plasmid containing human caspase-14 cDNA and the empty pCMV vector as control.

### Vector construction

The caspase-14 expression vector containing full-length cDNA of human caspase-14 in the pCMV6-XL4 plasmid was constructed and provided by OriGene (Rockville, MD). *Homo sapiens* caspase-14 cDNA (729 bp) was inserted into the Not I site of the pCMV6-XL4 plasmid. The construct was sequenced to confirm the cDNA sequence.

### Transfection

BRECs were transfected with the pCMV plasmid containing human caspase-14 cDNA and the empty pCMV vector using Lipofectamine-2000 Reagent (40 μg/ml; Invitrogen, Carlsbad, CA) according to the manufacturer’s instructions with some modification. BRECs were seeded in six-well plates (1×10^5^ cells/0.5 ml of growth medium). When cells reached 90%–95% confluence, growth medium was replaced with 3 ml of serum-free medium and transfected using a DNA (µg) to Lipofectamine-2000 ratio of 1:2 by diluting 2 μg of Lipofectamine-2000 in 50 µl of serum-free medium and 1 µg of DNA in 50 μl of serum-free medium. After incubation for 5 min at room temperature, the diluted DNA along with the diluted Lipofectamine-2000 reagent was combined, mixed gently, and incubated at room temperature for 20 min. About 100 µl of the complex was added to each well containing cells and medium, incubated at 37 °C in a CO_2_ incubator, and allowed to further grow overnight to complete confluency at which time cells were detached and seeded 1:10 in growth medium. Cells were treated with 250 µg/ml of Zeocin (Invitrogen) for selective treatment.

### Terminal deoxynucleotidyl transferase-mediated dUTP nick-end labeling assay

In situ cell death in retinal ECs transfected with the pCMV plasmid containing human caspase-14 cDNA and the empty pCMV vector as described above was evaluated using fluorescence labeled TUNEL assay (Roche, Indianapolis, IN). Cells were counted (75,000 cells/chamber), cultured in eight-chamber slides overnight, and transfected as described above. Two days later, cells were fixed with 4% paraformaldehyde in PBS, pH 7.4 for 1 h at room temperature, incubated in permeabilization solution (0.1% Triton X-100 in 0.1% sodium citrate) for 2 min on ice (2–8 °C), and rinsed twice with PBS. Then the cells were incubated in the TUNEL reaction mixture inside a humidified chamber for 60 min at 37 °C. Cells were then rinsed three times with PBS and mounted with antifade mounting medium containing DAPI (Vector Laboratories) for cell nuclei visualization. Apoptosis DNA fragmentation was visualized under florescence microscopy (Carl Zeiss Axiophot, Thornwood, NY), and representative pictures were taken at magnifications 40× and 20× from each chamber. The number of apoptotic cells was counted by randomly choosing a field of view and individually counting each apoptotic cell. DAPI was used as a reference to the nuclei.

Similarly, TUNEL staining of pericytes infected with adenovirus expressing caspase-14/GFP or GFP only was performed using the Click-iT TUNEL Alexa Fluor Imaging Assay (Invitrogen, Burlingame, CA) following the manufacturer’s manual. Positive cells were counted and calculated as a percentage of the total cell number.

### Statistical analysis

All data were summarized as means±SD. Statistical methods were used to compare different groups and determine the significance of the observed differences in all experiments. ANOVA followed by the Tukey post-hoc test was used to evaluate group differences. Results were considered significant when p<0.05. Experiments were performed at least three times to confirm reproducibility.

## Results

### Caspase-14 is expressed in different retinal cells

**Figure 1:**
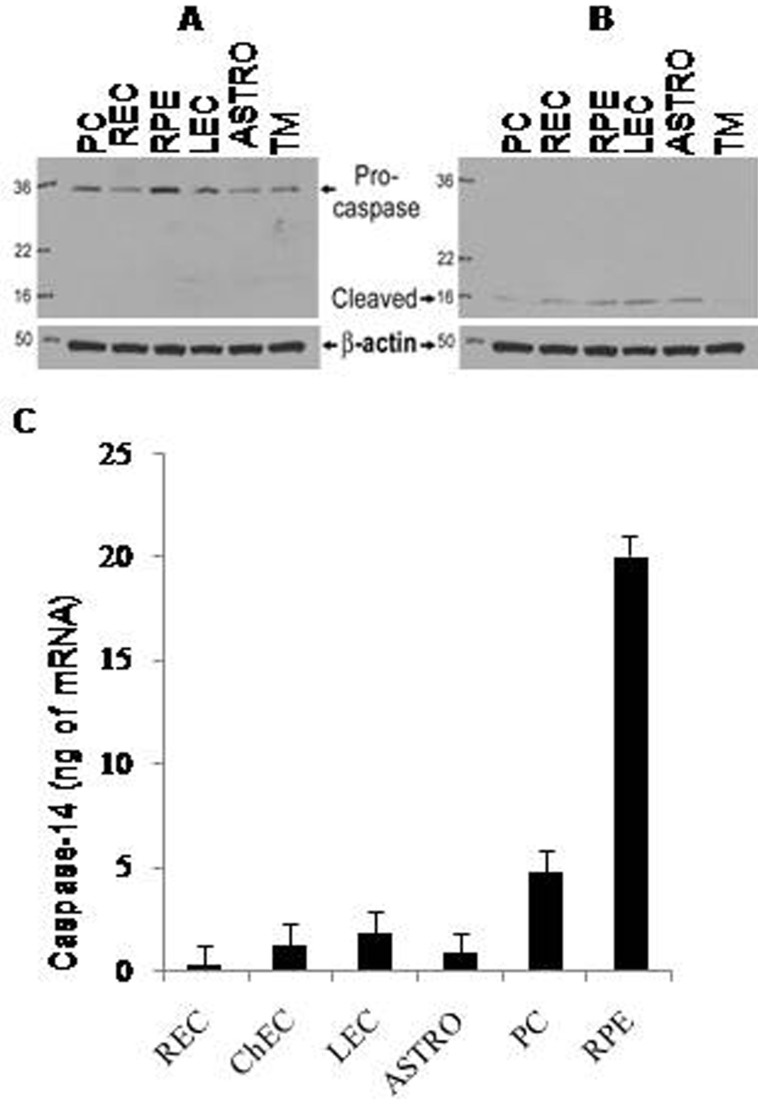
Expression of caspase-14 in the retinal cells. Western blot analysis of retinal cell lysates showed that caspase-14 is present (**A**) and is processed under reduced conditions in the presence of β-mercaptoethanol (**B**) in different retinal cells including pericytes (PCs), endothelial cells (RECs), pigment epithelium (RPE) cells, and astrocytes (ACs). Lung ECs and trabecular meshwork (TM) cells also expressed caspase-14. qPCR analysis of caspase-14 mRNA (**C**) demonstrated the presence of caspase-14 mRNA in the same cells and in choroidal ECs. Please note the higher expression of caspase-14 in PC and RPE cells compared to other retinal cells. The cell culture experiments were repeated using two different isolations with similar results.

We evaluated caspase-14 protein and gene expression in retinal PCs, ECs, ACs, ChECs, lung ECs, and RPE and TM cells. Western blotting and qPCR analysis showed that caspase-14 protein and mRNA are normally present in these cells and are processed in different retinal cells, with relatively higher amounts in retinal PCs and RPE cells compared to other cell types (Figure 1). These findings indicate that caspase-14 is expressed and can be processed in the retinal cells, and hence, exploring its role in ocular pathology is important.

### Diabetes increased caspase-14 expression in human and mouse retinas

**Figure 2:**
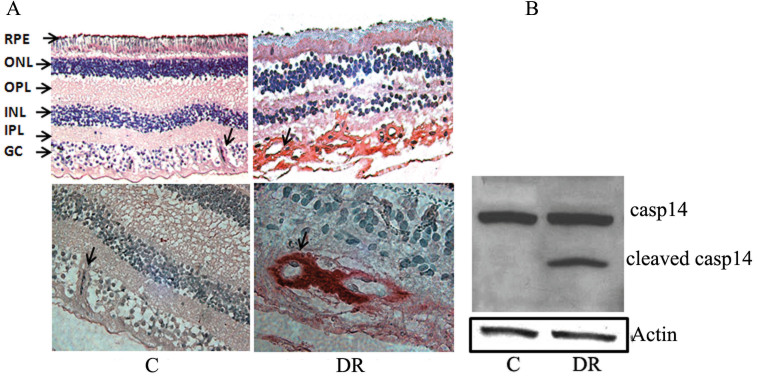
Caspase-14 in the human retina. Immunohistochemistry of caspase-14 showed upregulation of caspase-14 in the retinas of subjects with DR compared to the control (**A**). There was marked caspase-14 immunoreactivity in the retinal vascular and perivascular tissues of subjects with DR (arrows) compared to the retina of subjects without DR. Western blot analysis of caspase-14 in the retinas of human subjects showed a marked increase in the expression of caspase-14 and the processed caspase-14 in subjects with DR compared to the control (**B**).

To test whether caspase-14 is expressed in the retina and whether alterations occur with DR, we examined caspase-14 expression in retinas from diabetic and non-diabetic samples. We observed expression of caspase-14 in retinas with a marked increase in the retinal expression of caspase-14 in human subjects who had DR (Figure 2). Interestingly, we noticed the caspase-14 immunoreactivity was localized mainly to the retinal vasculatures (Figure 2A). Additionally, western blot analysis showed the presence of cleaved caspase-14 in the retinal homogenates of subjects with DR but not in subjects without DR (Figure 2B).

**Figure 3:**
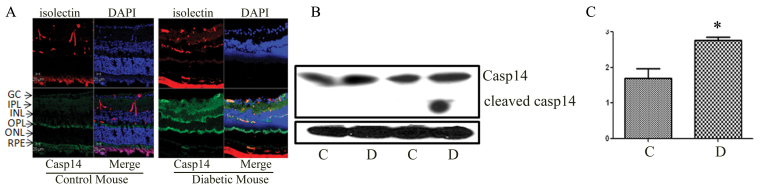
Caspase-14 in the mouse retina. Immunofluorescence reaction (**A**) using specific antibody against caspase-14 (green), vascular marker (isolectin B4-red), and nuclear stain (DAPI) showed marked increase in the expression of caspase-14 in different layers in particular in relation to retinal vasculatures (red). Western blot analysis of caspase-14 in the mouse retina (**B**) demonstrated significant upregulation in diabetic mice and the presence of cleaved caspase-14 in one of the diabetic mice compared to the control (**C**). (* p<0.05; D versus C; n=6).

We have previously reported increased leukostasis and vascular permeability in retinas of diabetic mice [26]. We next determined if these retinal microvascular changes were associated with changes in the retinal levels of caspase-14. Our experiments demonstrated a marked increase in caspase-14 immunoreactivity in the vascular and perivascular retinal tissues of diabetic mice compared to the control animals (Figure 3A). These observations were confirmed with western blot analysis of total caspase-14 in the retinal homogenate, which showed a significant increase in the expression of caspase-14 in the retinas of diabetic mice compared to the control group (Figure 3B,C; p<0.05). We also noticed the presence of cleaved caspase-14 in the retinas of some diabetic mice.

### Effect of high glucose on caspase-14 levels in retinal microvascular cells

**Figure 4:**
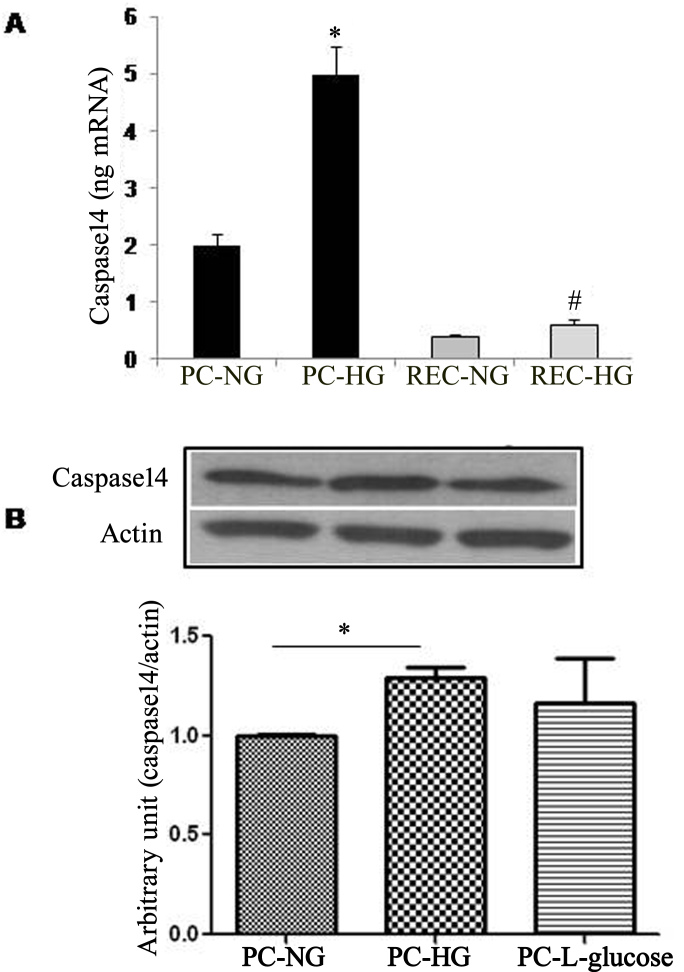
Effect of high glucose (HG) on caspase-14 expression in retinal endothelial cells (RECs) and pericytes (PCs). The qRT–PCR analysis of caspase-14 mRNA in retinal PCs and ECs showed a significant increase by HG compared with normal glucose (NG; **A**). The level of caspase-14 mRNA was relatively higher in retinal PCs under normal or high glucose conditions compared to retinal ECs. Western blot analysis of the caspase-14 protein level in PCs showed a significant increase by high glucose compared with normal glucose or L-glucose (**B**; * p<0.05).

Retinal PCs and ECs are crucial targets in the development and pathogenesis of DR. We next determined the effect of HG on caspase-14 mRNA expression in these cells. We observed a significant increase in the expression of caspase-14 mRNA under HG conditions, especially in retinal PCs, which demonstrated a higher level under normal and high glucose conditions compared to the retinal ECs (Figure 4A). The increase in caspase-14 mRNA in PCs was associated with a significant increase in the protein levels as determined with western blotting compared to the NG (p=0.024). The osmotic control L-glucose demonstrated a modest nonsignificant increase in the caspase-14 expression compared to the NG (p>0.05; Figure 4B).

### Effect of caspase-14 expression on retinal endothelial cells and pericytes

**Figure 5:**
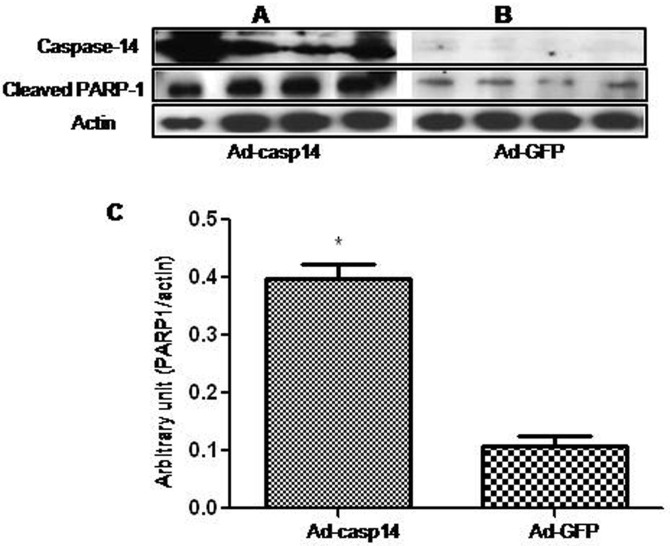
Effect of caspase-14 expression on the levels of cleaved PARP-1 in retinal ECs. Western blot analysis of caspase-14 in BRECs infected with adenovirus expressing caspase-14 and GFP (Ad-casp14; **A**) or GFP only (Ad-GFP; **B**) demonstrated overexpression of caspase-14 in Ad-casp14 compared to the Ad-GFP. Please note that overexpression of caspase-14 in BRECs was associated with a significant increase in the cleaved PARP-1 compared to the control cells infected with Ad-GFP (**C**; *p<0.05).

**Figure 6:**
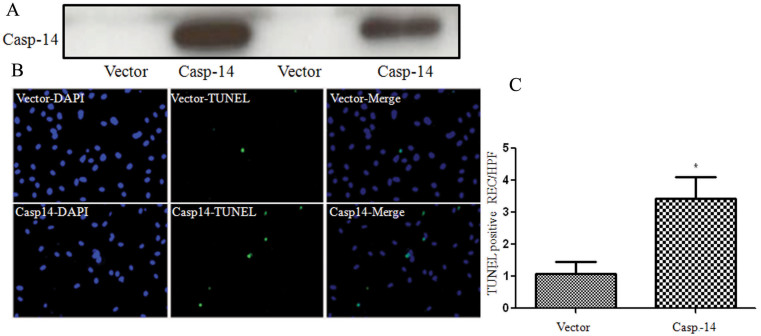
TUNEL staining of retinal EC. Retinal ECs were transfected with the pCMV plasmid containing human caspase-14 cDNA and the empty pCMV vector as a control (**A**). The number of apoptotic cells (green) was significantly increased in caspase-14 expressing pericytes compared to the control (**B**, **C**). DAPI (blue) is a nuclear staining (*p<0.05).

To evaluate whether caspase-14 is involved in the microvascular cell death in the retina during DR, we determined the effects of caspase-14 expression on retinal EC and PC survival. We first evaluated the impact of infecting BRECs with adenovirus encoding the full-length human caspase-14 cDNA and *GFP* cDNA or *GFP* cDNA only as a control. We noticed that expression of caspase-14 in BREC led to a significant upregulation of cleaved PARP-1 (Figure 5A), while GFP expression had no effect (Figure 5B). The quantitative assessment of the data are shown in Figure 5C. We next evaluated the rate of apoptosis in mouse retinal EC expressing caspase-14 with TUNEL assay. Our experiments demonstrated a significant increase in the number of apoptotic cells in retinal ECs expressing caspase-14 compared to the control (Figure 6).

**Figure 7:**
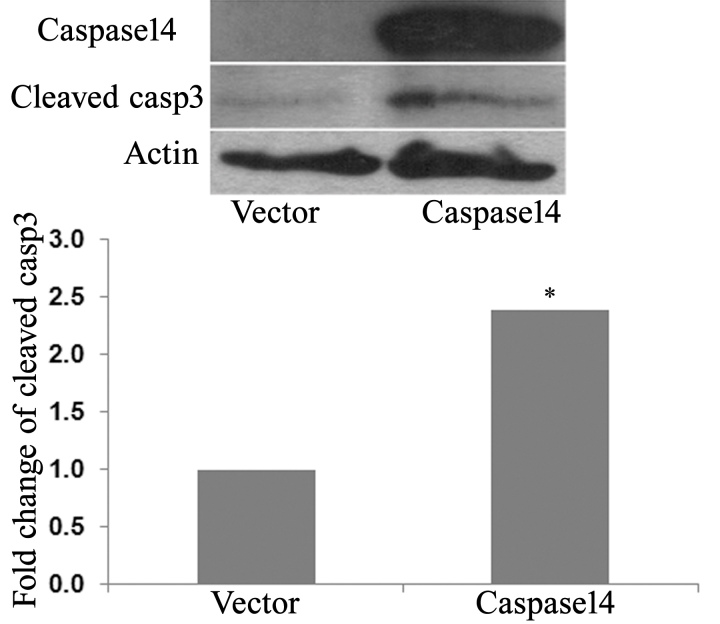
Western blot analysis of cleaved caspase-3 in retinal PCs. Pericytes (PCs) were infected with an adenovirus encoding the full-length human caspase-14 cDNA and green fluorescent protein (GFP) cDNA (Ad-casp14) or encoding only the GFP as a control (Ad-GFP). There was a nearly threefold increase in the expression of cleaved caspase-3 in caspase-14 expressing PCs compared to the control (*p<0.05).

**Figure 8:**
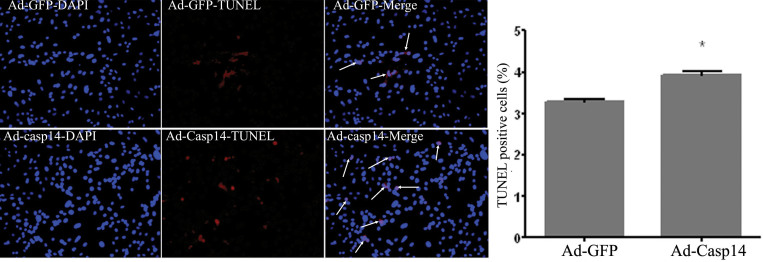
TUNEL staining of retinal PC expressing caspase 14. There was a significant increase in the number of apoptotic cells (arrows) in PC infected with caspase-14/GFP expressing adenovirus (Ad-casp14) compared to the PC infected with adenovirus expressing GFP (Ad-GFP) control (*p<0.05).

We next evaluated the impact of caspase-14 expression on retinal PCs by studying the changes in the levels of apoptotic markers and the number of apoptotic cells. Western blot analysis of cleaved caspase-3 demonstrated a significant increase in PCs infected with adenovirus encoding human caspase-14 cDNA and *GFP* cDNA compared to the PCs infected with the adenovirus encoding only the *GFP* as a control (Figure 7). Furthermore, the TUNEL assay of apoptosis showed a significant increase in the number of apoptotic cells in caspase-14 expressing PCs compared to the control cells (Figure 8). These results suggest that enhanced expression of caspase-14 under diabetic conditions may contribute to microvascular cell apoptosis and pathogenesis of DR.

## Discussion

Retinal microvascular cell loss is a characteristic feature of DR that occurs due to apoptosis [4,20]. However, the underlying biochemical and molecular mechanisms are not well understood. Our data suggest caspase-14 as a potential proapoptotic player in DR. To our knowledge, the current study is the first to demonstrate the detailed presence of caspase-14 in the retina and various retinal cells, and that alterations of caspase −14 may contribute to the development and progression of DR. The major findings were as follows: 1) expression of caspase-14 in the retina and various retinal cells, including retinal ECs, PCs, ACs, ChECs, and RPE and TM cells; 2) upregulation and activation of caspase-14 in the human and mouse retina during diabetes; 3) increased caspase-14 expression in retinal ECs and PCs cultured under high glucose conditions; and 4) an increased number of TUNEL-positive cells and levels of apoptotic markers, cleaved PARP-1 and caspase-3, in caspase-14 expressing retinal ECs and PCs.

Although the function of caspase-14 in different tissues is still to be elucidated, it is involved mainly in epithelial differentiation, which shares some features with apoptosis including DNA fragmentation, and nuclear condensation and activation of caspase-3 [11,27-29]. The proteolytic processing of procaspase-14 has been reported to increase in the brain following reperfusion injury, and this was linked to the increased number of neuronal cell deaths [14]. Similar observations have been reported in cardiac myocytes [13].

Apoptosis of retinal microvascular cells, PCs and ECs, is crucial for the development of acellular capillary, breakdown of the blood–retinal barrier, and subsequent neovascularization during diabetes [4,18,30-35]. Although the loss of microvascular cells is well established as an initial abnormality that can be morphologically detected in the early stages of DR, its pathogenesis is still poorly understood. Our data demonstrated that caspase-14 is normally produced by different cells involved in retinal vascular homeostasis, including retinal ECs, PCs, and ACs. The expression of caspase-14 and its processed form was increased in the human retinas of subjects with DR. Similar results were also observed in diabetic mice. We also observed that the PCs’ caspase-14 mRNA and protein levels were remarkably higher than those of the other retinal cells examined here and further increased under high glucose conditions. Moreover, our results demonstrated a marked increase in the number of TUNEL-positive cells with increased levels of apoptotic markers, cleaved poly [ADP-ribose] polymerase 1 (PARP-1) and caspase-3, in caspase-14 expressing ECs and PCs. Hence, altered production of caspase-14 may play a key role in the pathogenesis of retinal microvascular dysfunction during diabetes. Consistent with our results, a recent report demonstrated a marked increase in the amount of caspase-14 in aqueous humor of patients with glaucoma [15], supporting the presence of caspase-14 in the eye and its potential role in ocular diseases including DR in particular; glaucoma is also characterized by accelerated retinal neuronal cell death.

Interestingly, the enhanced apoptosis of retinal ECs and PCs that express caspase-14 occurred in the absence of caspase-14 cleavage. In agreement with other investigators [36], our data suggest that caspase-14 does not require cleavage for activity. Some caspases such as caspase-9 do not require cleavage for activity but rather require specific cofactors for enzymatic activity [37]. Hence, caspase-14 may function best in the presence of its required cofactors. One potential candidate that could be involved in activating caspase-14 is the calcium-regulated intracellular cysteine protease, calpain [36]. Calpain may provide a link between caspase-14 and pathogenesis of DR. Particularly, calpain inhibition has been reported to improve neovascular architecture and functional perfusion in ischemic retinopathy [38] and to preserve retinal function after transient retinal ischemia in rats [39].

Caspase-14 is also present in tissues or cells involved in the barrier function such as epidermal keratinocytes and RPE cells. We also observed caspase-14 expression in RPE cells and choroidal ECs, perhaps impacting choroidal vascular function and the outer-retinal barrier. Caspase-14 protein expression is detected in the cytoplasm of the RPE cells in embryonic and adult mouse and human samples [12]. RPE cells play a significant role in ocular vascular homeostasis by producing pro- and antiangiogenic factors [40]. We have recently shown the exogenous expression of caspase-14 in tumor cells blocks tumor growth through inhibition of angiogenesis [41]. Thus, production of caspase-14 by RPE cells may contribute to the integrity of the RPE layer and function, and prevent choroidal vessel growth into the retina.

The next challenge is to identify how caspase-14 expression and activity are regulated in the retina. A recent report suggested that p38 and c-Jun N-terminal kinase mitogen-activated protein kinase pathways are essential for caspase-14 expression in normal human epidermal keratinocytes [42]. In retina, p38 mitogen-activated protein kinase plays a crucial role in the pathogenesis of DR via inflammatory and proapoptotic cell death pathways [43,44]. Further investigation is needed to delineate fully the signal transduction pathways involved in regulating caspase-14 expression and/or activity and function during DR.

In summary, we demonstrate that caspase-14 is present in the retina and various retinal cells with an important role in maintaining normal retinal vascular homeostasis. Moreover, human and experimental DR was associated with upregulation and processing of caspase-14, and high glucose resulted in significant increases in caspase-14 levels in cultured PCs and retinal ECs. Caspase-14 overexpression in retinal ECs and PCs resulted in upregulation of cleaved PARP-1 and caspase-3 and a significant increase in the number of apoptotic cells. In conclusion, activation of caspase-14 under pathological conditions may influence retinal vascular function by promoting apoptosis of retinal microvascular cells. Further studies are required to determine the mechanisms of caspase-14 regulation and its coordinated interactions with other caspases.
